# Dr. Brown Sequard

**Published:** 1864-11

**Authors:** 


Dr. Brown Sequard.—We notice by the *Boston Medical & Surgical Journal* that this distinguished gentleman will be prevented by ill health from delivering his course of lectures contemplated in connection with the course of the Boston Medical College this winter.

